# Supplementary material for: Chlorhexidine and octenidine susceptibility of bacterial isolates from clinical samples in a three-armed cluster randomised decolonisation trial
Source: PLoS One. 2022 Dec 14;17(12):e0278569. doi: 10.1371/journal.pone.0278569 (PMC9749986; doi:10.1371/journal.pone.0278569)
Supplement: S1 Table — (DOCX) [file pone.0278569.s002.docx]

**Supplemental material**

**S1 Table: Reference strains with minimal inhibitory concentrations (µg/ml) to chlorhexidine and octenidine.**

|  | **Chlorhexidine** |  | **Octenidine** |  |
| --- | --- | --- | --- | --- |
| **Reference strain** | **MIC in [%] of stock solution (concentrated 20.000 µg / ml)** | **MIC in [µg / ml]** | **MIC in [%] of stock solution**  **(concentrated 800 µg / ml)** | **MIC in [µg / ml]** |
| ***Staphylococcus aureus* ATCC 6538** | 0.01% | 2 µg/ml | 0.5% | 4.0 µg/ml |
| ***Escherichia coli* NCTC 10538** | 0.01% | 2 µg/ml | 0.5% | 4.0 µg/ml |
| ***Klebsiella pneumoniae* ESBL DSM 16609** | 0.25% | 50 µg/ml | 0.5% | 4.0 µg/ml |
| ***Proteus mirabilis* ATCC 14153** | 0.25% | 50 µg/ml | 0.5% | 4.0 µg/ml |
| ***Pseudomonas aeruginosa* ATCC 15442** | 0.5% | 100 µg/ml | 3% | 24.0 µg/ml |
