# Supplementary material for: Chlorhexidine and octenidine susceptibility of bacterial isolates from clinical samples in a three-armed cluster randomised decolonisation trial
Source: PLoS One. 2022 Dec 14;17(12):e0278569. doi: 10.1371/journal.pone.0278569 (PMC9749986; doi:10.1371/journal.pone.0278569)
Supplement: S2 Table — Median MIC (MIC50) with inter quartile ranges (IQR) and MIC90 are given in in [%] of extraction solution and are reported for Staphylococcus aureus, coagulase-negative staphylococci, Escherichia coli, Klebsiella spp. and Pseudomonas aeruginosa stratified by study group (chlorhexidine, octenidine, routine care) and period (baseline = prior to the intervention period, intervention = at the end of the intervention period). (DOCX) [file pone.0278569.s003.docx]

**S2 Table: Chlorhexidine and octenidine minimal inhibitory concentrations (MIC) of bacterial isolates** **from clinical samples in percentage of extraction solution.** Median MIC (MIC_50_) with inter quartile ranges (IQR) and MIC_90_ are given in in [%] of stock solution and are reported for *Staphylococcus aureus*, coagulase-negative staphylococci*, Escherichia coli*, *Klebsiella* spp. and *Pseudomonas aeruginosa* stratified by study group (chlorhexidine, octenidine, routine care) and period (baseline = prior to the intervention period, intervention = at the end of the intervention period).

|  | **Total** | **Chlorhexidine group** | | **Octenidine group** | | **Routine care group = control** | |
| --- | --- | --- | --- | --- | --- | --- | --- |
|  |  | **Baseline** | **Intervention** | **Baseline** | **Intervention** | **Baseline** | **Intervention** |
| ***Staphylococcus aureus*** |  |  |  |  |  |  |  |
| n (%) | 155 (100%) | 38 (25.0%) | 22 (14.1%) | 24 (15.4%) | 18 (11.5%) | 23 (14.7%) | 30 (19.2%) |
| **Chlorhexidine** |  |  |  |  |  |  |  |
| MIC_50_ (IQR) in [%] of stock solution | 0.025 (0.01-0.025) | 0.025(0.025-0.025) | 0.010(0.010- 0.022) | 0.025(0.01-0.025) | 0.010(0.01- 0.025) | 0.025(0.010-0.025) | 0.025(0.014-0.050) |
| MIC_90_ in [%] of stock solution | 0.025 | 0.025 | 0.022 | 0.025 | 0.025 | 0.025 | 0.050 |
| **Octenidine** |  |  |  |  |  |  |  |
| MIC_50_ in [%] of stock solution (IQR) | 0.25(0.25-0.25) | 0.25 (0.25-0.50) | 0.25 (0.1-0.25) | 0.25 (0.25-0.25) | 0.25 (0.25-0.25) | 0.25 (0.25-0.50) | 0.25 (0.25-0.25) |
| MIC_90_ in [%] of stock solution | 0.25 | 0.50 | 0.25 | 0.25 | 0.25 | 0.50 | 0.25 |
| **Coagulase-negative staphylococci** |  |  |  |  |  |  |  |
| n (%) | 122 (100%) | 31 (25.4%) | 31 (25.4%) | 17 (13.9%) | 11 (9.0%) | 15 (12.3%) | 17 (13.9%) |
| **Chlorhexidine** |  |  |  |  |  |  |  |
| MIC_50_ in [%] of stock solution (IQR) | 0.025(0.01-0.025) | 0.025(0.01-0.025) | 0.01(0.005- 0.01) | 0.025(0.025-0.025) | 0.01(0.005- 0.018) | 0.025(0.01- 0.025) | 0.025(0.0025- 0.025) |
| MIC_90_ in [%] of stock solution | 0.025 | 0.025 | 0.01 | 0.025 | 0.018 | 0.025 | 0.025 |
| **Octenidine** |  |  |  |  |  |  |  |
| MIC_50_ in [%] of stock solution (IQR) | 0.25(0.25-0.50) | 0.25 (0.25-0.50) | 0.25 (0.10-0.25) | 0.25 (0.25-0.50) | 0.10 (0.10-0.25) | 0.50 (0.25-0.50) | 0.25 (0.25-0.25) |
| MIC_00_ in [%] of stock solution | 0.50 | 0.50 | 0.250 | 0.50 | 0.25 | 0.50 | 0.25 |
| ***Escherichia coli*** |  |  |  |  |  |  |  |
| n (%) | 227 (100%) | 64 (28.2%) | 37 (16.3%) | 41 (18.1%) | 18 (7.9%) | 39 (17.2%) | 28 (11.9%) |
| **Chlorhexidine** |  |  |  |  |  |  |  |
| MIC_50_ in [%] of stock solution (IQR) | 0.025(0.01-0.025) | 0.025(0.01-0.025) | 0.01(0.01- 0.025) | 0.025(0.025-0.025) | 0.025(0.01- 0.025) | 0.025(0.025-0.025) | 0.025(0.01-0.05) |
| MIC_90_ in [%] of stock solution | 0.025 | 0.025 | 0.025 | 0.025 | 0.025 | 0.025 | 0.05 |
| **Octenidine** |  |  |  |  |  |  |  |
| MIC_50_ in [%] of stock solution (IQR) | 0.5(0.25-0.5) | 0.50 (0.5-0.5) | 0.25 (0.25-0.50) | 0.50 (0.50-0.50) | 0.25 (0.25-0.50) | 0.50 (0.50-0.50) | 0.50 (0.25-0.5) |
| MIC_90_ in [%] of stock solution | 0.50 | 0.50 | 0.50 | 0.50 | 0.50 | 0.50 | 0.50 |
| ***Klebsiella spp.*** |  |  |  |  |  |  |  |
| n (%) | 150 (100%) | 28 (18.7%) | 28 (18.7%) | 23 (15.3%) | 29 (19.3%) | 20 (13.3%) | 22 (14.7%) |
| **Chlorhexidine** |  |  |  |  |  |  |  |
| MIC_50_ in [%] of stock solution (IQR) | 0.25(0.10-0.25) | 0.25(0.10-0.25) | 0.10(0.10- 0.25) | 0.25(0.25-0.25) | 0.25(0.10-0.25) | 0.25(0.21-0.25) | 0.25(0.10-0.25) |
| MIC_90_ in [%] of stock solution | 0.25 | 0.25 | 0.25 | 0.25 | 0.25 | 0.25 | 0.25 |
| **Octenidine** |  |  |  |  |  |  |  |
| MIC_50_ in [%] of stock solution (IQR) | 0.50(0.50-1.00) | 0.50 (0.50-1.5) | 0.50 (0.50-0.50) | 1.00 (0.50-1.25) | 0.50 (0.50-0.50) | 0.50 (0.50-1.63) | 0.50 (0.50-0.50) |
| MIC_90_ in [%] of stock solution | 1.00 | 1.5 | 0.5 | 1.25 | 0.5 | 1.63 | 0.5 |
| ***Pseudomonas aeruginosa*** |  |  |  |  |  |  |  |
| n (%) | 136 (100%) | 19 (14.0%) | 18 (13.2%) | 17 (12.5%) | 40 (29.4%) | 18 (13.2%) | 24 (17.6%) |
| **Chlorhexidine** |  |  |  |  |  |  |  |
| MIC_50_ in [%] of stock solution (IQR) | 0.25(0.25-0.25) | 0.25(0.18-0.25) | 0.25(0.25-0.25) | 0.25(0.25-0.25) | 0.25(0.25-0.25) | 0.25(0.25-0.25) | 0.25(0.25-0.25) |
| MIC_90_ in [%] of stock solution | 0.25 | 0.25 | 0.25 | 0.25 | 0.25 | 0.25 | 0.25 |
| **Octenidine** |  |  |  |  |  |  |  |
| MIC_50_ in [%] of stock solution (IQR) | 1.50(1.00-3.00) | 1.00 (1.00-2.50) | 1.00 (1.00-2.00) | 2.00 (1.00-2.00) | 1.50 (1.00-2.00) | 3.00 (1.00-4.00) | 2.00 (1.00-3.00) |
| MIC_90_ in [%] of stock solution | 3.00 | 2.50 | 2.00 | 16.00 | 2.00 | 4.00 | 3.00 |
