# Supplementary material for: Chlorhexidine and octenidine susceptibility of bacterial isolates from clinical samples in a three-armed cluster randomised decolonisation trial
Source: PLoS One. 2022 Dec 14;17(12):e0278569. doi: 10.1371/journal.pone.0278569 (PMC9749986; doi:10.1371/journal.pone.0278569)
Supplement: S3 Table — (DOCX) [file pone.0278569.s004.docx]

**S3 Table: Characteristics of *S. aureus* isolates from clinical samples with chlorhexidine MIC ≥ 8µg/ml.**

| **Isolate** | **ICU** | **ICU day of sample collection** | **Sample site** | **Study group** | **Study period** | **Chlorhexidine MIC** in est. [µg/ml] | **Octenidine MIC** in est. [µg/ml] |
| --- | --- | --- | --- | --- | --- | --- | --- |
| #1 | 1 | 6 | Tracheal aspirate | Octenidine | Intervention | 10.0 | 4.0 |
| #2 | 1 | 4 | Tracheal aspirate | Octenidine | Intervention | 10.0 | 0.8 |
| #3 | 3 | 74 | Wound | Chlorhexidine | Baseline | 15.0 | 2.0 |
| #4 | 5 | 24 | Blood | Control | Baseline | 15.0 | 4.0 |
| #5 | 5 | 4 | Tracheal aspirate | Control | Intervention | 10.0 | 2.0 |
| #6 | 14 | 21 | Tracheal aspirate | Control | Baseline | 15.0 | 2.0 |
| #7 | 14 | 5 | Tracheal aspirate | Control | Intervention | 50.0 | 4.0 |
| #8 | 14 | 13 | Tracheal aspirate | Control | Intervention | 10.0 | 4.0 |
| #9 | 14 | 62 | Tracheal aspirate | Control | Intervention | 10.0 | 2.0 |
| #10 | 15 | 3 | Tracheal aspirate | Chlorhexidine | Baseline | 15.0 | 6.0 |
| #11 | 15 | 4 | Tracheal aspirate | Chlorhexidine | Baseline | 50.0 | 2.0 |
| #12 | 31 | 20 | Tracheal aspirate | Chlorhexidine | Baseline | 20.0 | 6.0 |
| #13 | 31 | 30 | Urine | Chlorhexidine | Baseline | 10.0 | 4.0 |
| #14 | 36 | 4 | Wound | Control | Intervention | 10.0 | 2.0 |
| #15 | 36 | 3 | Wound | Control | Intervention | 10.0 | 2.0 |
| #16 | 36 | 3 | Tracheal aspirate | Control | Intervention | 10.0 | 2.0 |
| #17 | 51 | 5 | Tracheal aspirate | Control | Intervention | 20.0 | 4.0 |
| #18 | 51 | 7 | Tracheal aspirate | Control | Intervention | 10.0 | 2.0 |
| #19 | 51 | 8 | Tracheal aspirate | Control | Intervention | 10.0 | 2.0 |
| #20 | 55 | 13 | Tracheal aspirate | Chlorhexidine | Baseline | 50.0 | 16.0 |
| #21 | 60 | 9 | Blood | Control | Intervention | 20.0 | 2.0 |
| #22 | 64 | 4 | Wound | Control | Baseline | 50.0 | 4.0 |
| #23 | 64 | 4 | Urine | Control | Intervention | 10.0 | 2.0 |
