# Supplementary material for: Chlorhexidine and octenidine susceptibility of bacterial isolates from clinical samples in a three-armed cluster randomised decolonisation trial
Source: PLoS One. 2022 Dec 14;17(12):e0278569. doi: 10.1371/journal.pone.0278569 (PMC9749986; doi:10.1371/journal.pone.0278569)
Supplement: S4 Table — Chlorhexidine and octenidine susceptibility of bacterial isolates were reported as binary variable (chlorhexidine / octenidine MIC of bacterial isolates ≥ species-specific chlorhexidine/ octenidine MIC50 (yes / no). aP-values were reported for comparisons between chlorhexidine/ octenidine MIC50 ≥ species-specific chlorhexidine/ octenidine MIC50 = “yes” or “no”. b percentage of columns. c percentage of rows. P-values < 0.05 were interpreted as significant (*). n, number. (%), percent. (DOCX) [file pone.0278569.s005.docx]

**S4 Table: Susceptibility of ICU-attributable bacterial isolates from clinical samples (n = 790) to chlorhexidine and octenidine (all species) stratified by chlorhexidine and octenidine susceptibility.** Chlorhexidine and octenidine susceptibility of bacterial isolates were reported as binary variable (chlorhexidine / octenidine MIC of bacterial isolates ≥ species-specific chlorhexidine/ octenidine MIC_50_ (yes / no). ^a^P-values were reported for comparisons between chlorhexidine/ octenidine MIC50 ≥ species-specific chlorhexidine/ octenidine MIC_50_ = “yes” or “no”. ^b^ percentage of columns. ^c^ percentage of rows. P-values < 0.05 were interpreted as significant (*). n, number. (%), percent.

| **Chlorhexidine** | **Total** | **Chlorhexidine MIC ≥ species-specific chlorhexidine and octenidine MIC_50_** |  |  |
| --- | --- | --- | --- | --- |
|  |  | **yes** | **no** | **P-value^a^** |
| **All, n (%^b^;%^c^)** | **790 (100.0; 100.0)** | **537 (100.0; 68.0)** | **253 (100.0; 32.0)** |  |
| **Study group and period** |  |  |  |  |
| Chlorhexidine group in the baseline period, n (%^b^;%^c^) | 180 (22.8; 100.0) | 126 (23.4; 70.0) | 54 (21.3; 30.0) | 0.507 |
| Chlorhexidine group in the intervention period, n (%^b^;%^c^) | 136 (17.2; 100.0) | 59 (11.0; 43.4) | 77 (30.4; 56.6) | <0.001* |
| Octenidine group in the baseline period, n (%^b^;%^c^) | 122 (15.4; 100.0) | 102 (19.0; 83.6) | 20 (7.9; 16.4) | < 0.001* |
| Octenidine group in the intervention period, n (%^b^;%^c^) | 116 (14.7; 100.0) | 77 (14.3; 66.4) | 39 (15.4; 33.6) | 0.690 |
| Control group in the baseline period, n (%^b^;%^c^) | 115 (14.6; 100.0) | 85 (15.8; 73.9) | 30 (11.9; 26.1) | 0.140 |
| Control group in the intervention period, n (%^b^;%^c^) | 121 (15.3; 100.0) | 88 (16.4; 72.7)) | 33 (13.0; 27.3) | 0.223 |
| **ICU day** of sample collection, median (IQR) | 9 (5 – 18) | 10 (5 – 20) | 8 (5 – 16) | 0.059 |
| **Clinical material** |  |  |  |  |
| Blood, n (%^b^;%^c^) | 185 (23.4; 100.0) | 99 (18.4; 53.5) | 86 (34.0; 46.5) | < 0.001* |
| Tracheal aspirate, n (%^b^;%^c^) | 330 (41.8; 100.0) | 234 (43.6; 70.9) | 96 (37.9; 29.1) | 0.134 |
| Urine, n (%^b^;%^c^) | 87 (11.0; 100.0) | 62 (11.5; 71.3) | 25 (9.9; 28.7) | 0.486 |
| Wound, n (%^b^;%^c^) | 87 (11.0; 100.0) | 63 (11.7; 72.4) | 24 (9.5; 27.6) | 0.347 |
| Others, n (%^b^;%^c^) | 101 (12.8; 100.0) | 79 (14.7; 78.2) | 22 (8.7; 21.8) | 0.018* |
|  |  |  |  |  |
| **Octenidine** |  | **Octenidine MIC ≥ species-specific chlorhexidine and octenidine MIC_50_** |  |  |
|  |  | **yes** | **no** | **P-value^a^** |
| **All, n (%^b^;%^c^)** | **790 (100.0; 100.0)** | **623 (100.0; 78.9)** | **167 (100.0; 21.1))** |  |
| **Study group and period** |  |  |  |  |
| Chlorhexidine group in the baseline period, n (%^b^;%^c^) | 180 (22.8; 100.0) | 148 (23.8; 82.2) | 32 (19.2; 17.8) | 0.209 |
| Chlorhexidine group in the intervention period, n (%^b^;%^c^) | 136 (17.2; 100.0) | 89 (14.3; 65.4) | 47 (28.1; 34.6) | <0.001* |
| Octenidine group in the baseline period, n (%^b^;%^c^) | 122 (15.4; 100.0) | 111 (17.6; 91.0) | 11 (17.8; 9.0) | < 0.001* |
| Octenidine group in the intervention period, n (%^b^;%^c^) | 116 (14.7; 100.0) | 82 (13.2; 70.7) | 34 (20.4; 29.3) | 0.020* |
| Control group in the baseline period, n (%^b^;%^c^) | 115 (14.6; 100.0) | 98 (15.7; 85.2) | 17 (10.2; 14.8) | 0.071 |
| Control group in the intervention period, n (%^b^;%^c^) | 121 (15.3; 100.0) | 95 (15.2; 78.5) | 26 (15.6; 21.5) | 0.714 |
| **ICU day** of sample collection, median (IQR) | 9 (5 – 18) | 10 (5 – 19) | 9 (4 – 17) | 0.274 |
| **Clinical material** |  |  |  |  |
| Blood, n (%^b^;%^c^) | 185 (23.4; 100.0) | 150 (24.1; 81.1) | 35 (21.0; 18.9) | 0.398 |
| Tracheal aspirate, n (%^b^;%^c^) | 330 (41.8; 100.0) | 259 (41.6; 78.5) | 71 (42.5; 21.5) | 0.827 |
| Urine, n (%^b^;%^c^) | 87 (11.0; 100.0) | 63 (10.1; 72.4) | 24 (14.4; 27.6) | 0.118 |
| Wound, n (%^b^;%^c^) | 87 (11.0; 100.0) | 69 (11.1; 79.3) | 18 (10.8; 20.7) | 0.913 |
| Others, n (%^b^;%^c^) | 101 (12.8; 100.0) | 82 (13.2; 81.2) | 19 (11.4; 18.8) | 0.540 |
