# Supplementary material for: Chlorhexidine and octenidine susceptibility of bacterial isolates from clinical samples in a three-armed cluster randomised decolonisation trial
Source: PLoS One. 2022 Dec 14;17(12):e0278569. doi: 10.1371/journal.pone.0278569 (PMC9749986; doi:10.1371/journal.pone.0278569)
Supplement: Attachment — Submitted filename: PONE_D-22-18018_R1_Rebuttal_final.docx [file pone.0278569.s007.docx]

Dear editor, dear reviewers,

Thank you very much for your thorough and helpful comments and advises on our manuscript PONE-D-22-18018 entitled “Chlorhexidine and octenidine susceptibility of bacterial isolates from clinical samples in a three-armed cluster randomised decolonisation trial”. Please find our point by point response below and our revised version of the manuscript PONE-D-22-18018_R1 uploaded. All lines reported here refer to the manuscript with track changes.

Comments by editor and reviewers are marked in grey with black font, responses by the authors in blue fonts without marks and changes in the manuscript were marked in yellow with black font.

We hope that we now fulfill all journal requirements, that we could answer all open questions to your full satisfaction and that we could dissolve all your concerns. Please do not hesitate to contact us, if you need any further information or have any further questions. Thank you very much in advance.

Kind Regards,

Luisa Denkel

When submitting your revision, we need you to address these additional requirements.

1. Please ensure that your manuscript meets PLOS ONE's style requirements, including those for file naming. The PLOS ONE style templates can be found at

https://journals.plos.org/plosone/s/file?id=wjVg/PLOSOne_formatting_sample_main_body.pdf and

<https://journals.plos.org/plosone/s/file?id=ba62/PLOSOne_formatting_sample_title_authors_affiliations.pdf>

Thank you for your advice. We revised the manuscript accordingly. We hope that our manuscript meets all PLOS ONE’s style requirements now.

2. Thank you for stating the following in the Acknowledgments Section of your manuscript:

"We thank all infection control practitioners, nurses, physicians, healthcare staff and technicians from all ICUs and laboratories participating in and supporting the CLIP-ID trial. We are grateful to Sage Products 17/ Stryker and Schülke as manufacturers of the (antiseptic) products applied in this trial, for their support. We thank Dr. Brill + Partner GmbH, Hamburg, Germany for their contract work."

We note that you have provided funding information that is not currently declared in your Funding Statement. However, funding information should not appear in the Acknowledgments section or other areas of your manuscript. We will only publish funding information present in the Funding Statement section of the online submission form.

Please remove any funding-related text from the manuscript and let us know how you would like to update your Funding Statement. Currently, your Funding Statement reads as follows:

"Funding for the CLIP-ID trial was provided by the German Federal Ministry of Education and Research within the scope of the InfectControl consortium (03ZZ0807A). Sage Products / Stryker and Schülke funded the (antiseptic) products for the intervention and supported the investigation of tolerances to chlorhexidine and octenidine by an independent accredited contract laboratory. The German Ministry of Education and Research (funder) and companies (Sage Products / Stryker, Schülke) funding products and the investigation of tolerances to chlorhexidine and octenidine by an independent laboratory had no role in study design, data collection and analysis, data interpretation, the decision to publish, or the preparation of the manuscript."

As requested, we revised the funding statement as follows:

"Funding for the CLIP-ID trial was provided by the German Federal Ministry of Education and Research within the scope of the InfectControl consortium (03ZZ0807A). Sage Products (Cary, USA) / Stryker (Kalamazoo, USA) and Schülke (Norderstedt, Germany) funded the (antiseptic) products for the interventions and supported the investigation of tolerances to chlorhexidine and octenidine by an independent accredited contract laboratory (Dr. Brill + Partner GmbH, Hamburg, Germany). The German Ministry of Education and Research (funder) and companies (Sage Products / Stryker, Schülke, Dr. Brill + Partner GmbH) had no role in study design, data collection and analysis, data interpretation, the decision to publish, or the preparation of the manuscript."

Please include your amended statements within your cover letter; we will change the online submission form on your behalf.

We included the revised (final) funding statement in the revised cover letter.

3. Please include captions for your Supporting Information files at the end of your manuscript, and update any in-text citations to match accordingly. Please see our Supporting Information guidelines for more information: http://journals.plos.org/plosone/s/supporting-information.

Thank you for this comment. We changed Table captions from “Table S1” to “S1 Table” in the supporting information file, in the supporting information captions (line 533ff) and the throughout the entire manuscript.

4. We note that the original protocol that you have uploaded as a Supporting Information file contains an institutional logo. As this logo is likely copyrighted, we ask that you please remove it from this file and upload an updated version upon resubmission.

We uploaded a new version of the original protocol without any institutional logos (S1 file).

Additional Editor Comments:

The manuscript was reviewed by two reviewers with expertise in this area and a statistical reviewer. Please note their comments below with which I agree. Specifically,

5. Please provide the rationale for use of and methods used to determine of the concentration of antiseptic solution generated from wringing out wipes (vs using stock solution)

Thank you for this important comment. This request is identical to comment 1 raised by reviewer 1. Please also see our response to that below. The manuscript has been revised accordingly. To avoid confusion, we used the term “extraction solutions” instead of “stock solutions” for the chlorhexidine / octenidine solutions harvested by wringing out the ready-to-use-products.

We revised all sections of the manuscript accordingly:

Methods’ section: (line 113 ff).

“Extraction solutions of antiseptic substances (chlorhexidine and octenidine) were harvested by wringing out chlorhexidine-impregnated cloths (Stryker / Sage Products) and octenidine wash mitts (Schülke) provided by the manufacturer. Concentrations of these extraction solutions were estimated to be similar to the concentrations of the ready-to-use products reported by the manufacturers: 2% (20,000 µg/ml) for chlorhexidine and 0.08% (800 µg/ml) for octenidine. Dilutions of antiseptic substances were made in water of standardized water hardness (WSH) according to VAH method 5 [1]. Briefly, bacteria were inoculated in soybean casein digest agar (CSA) at 37°C for 18 – 24h. Concentrations of these bacterial solutions were adjusted to 1.5 – 5.0 x 10^8^ colony forming units (CFU)/ml. Subsequently, 150 µl of the respective antiseptic dilutions and 150 µl doubled concentrated soybean casein solutions (CSL) were mixed. Microtiter plates were inoculated with 3 µl bacteria (1:10-dilution in CSL) yielding in a final bacterial concentration of 1.5 – 5.0 x 10^5^ colony forming units (CFU)/ml. Bacteria were inoculated at 37°C for 48 h. The lowest concentration of extraction solutions without bacterial growth (detectable turbidity) was interpreted as minimal inhibitory concentration (MIC) reported in percentage of extraction solution. Percentages were converted to estimated (est.) concentrations of extraction solutions in µg / ml. Controls were inoculated with WSH instead of antiseptic dilutions. Determination of bacteriostatic efficacy and suitable neutralizing agents was done according to VAH method 7 [1]. The following reference strains were included to the analysis: *Staphylococcus aureus* ATCC6538 (DSM 799), *Proteus mirabilis* ATCC 14153 (DSM 788), *Klebsiella pneumoniae* ESBL (DSM 16609), *Pseudomonas aeruginosa* ATCC 15442 and *Escherichia coli* NCTC 10538.”

Further, we revised the discussion (line 366 ff).

“Further, MIC tests were not performed with pure chlorhexidine and octenidine substances but with extraction solutions harvested by wringing out the antiseptic products. Pure stock solutions were not provided by the manufacturers. In consequence, MIC reported here could only be estimated from the concentrations reported for the antiseptic products (2µg / ml for chlorhexidine and 0.08 µg/ml of octenidine) and the dilutions (MIC in percentages) derived from of our extraction solutions. […]. Further, this approach might consider fractions of chlorhexidine and octenidine remaining in the cloths / mitts during the bathing process. This would not be factored in when using pure stock solutions. Thus, we consider our approach being appropriate and even more conservative. However, our estimated MIC might be overestimated and true MICs of bacterial isolates from clinical samples to chlorhexidine and octenidine might be lower.”

6. As pointed out by the statistical reviewer, provide power calculations on the effect size for differences in antiseptic susceptibility that could be detected with the target sample size that was already set based on the primary clinical outcome of the trial. The use of more appropriate statistical models is also suggested to statistically compare differences between groups over time.

Thank you for this important comment. As requested by the editor and the statistical reviewer, we added more statistical models to increase the quality of our manuscript. Further, we performed a power calculation. The manuscript has been revised accordingly in methods (line 162ff), results (line 252ff, Table 3, S4 Table) and discussion (323 ff and 346f).

Methods (line 162ff): “However, we performed a power calculation for the outcomes chlorhexidine or octenidine MIC being ≥ the species-specific chlorhexidine / octenidine MIC_50_. The latter were identified in this study (Table 2). We assumed that 35% of isolates per 100 patients isolates were ≥ MIC_50_ in the control group (routine care) compared to 65 % in the intervention group (antiseptic bathing with chlorhexidine or octenidine). Further, we assumed a ratio of 1:1 between the groups. With individual randomization, a sample size of 70 bacterial isolates in each group would have been required to show the difference with a power of 80%, and a two-sided type 1 error of 0.025. Adjusting for cluster effects with 13 clusters per arm and an intracluster correlation coefficient of 0.1 (adapted from literature [2]), we must have included 101 bacterial isolates per arm. Multiple comparisons to the routine care group were considered by a two-sided type 1 error of 0.025. “

Results: Please see line 252ff, Table 3 and S4 Table. “Characteristics of bacterial isolates (all species) stratified by chlorhexidine or octenidine MIC ≥ species-specific MIC50 (yes / no) are shown in S4 Table.” We used generalized estimated equations models to calculate adjusted incidence rate ratios (aIRR). Please see Table 3 and line 252ff for results: “ We used GEE models to investigate the impact of antiseptic bathing with chlorhexidine or octenidine on the chlorhexidine and octenidine susceptibility of bacterial isolates (all species). Characteristics of bacterial isolates (all species) stratified by chlorhexidine or octenidine MIC ≥ species-specific MIC50 (yes / no) are shown in S4 Table. aIRR for the chlorhexidine and the octenidine models are depicted in Table 3. The proportion of bacterial isolates with MIC ≥ species-specific chlorhexidine and octenidine MIC_50_ did not significantly differ between chlorhexidine or octenidine intervention periods compared with the control group (intervention period, Table 3). We detected significant differences between study groups and study periods with our GEE models (Table 3). However, these differences seemed to be independent from antiseptic bathing. In fact, aIRRs showed that the proportions of bacterial isolates with chlorhexidine / octenidine MIC ≥ species-specific chlorhexidine / octenidine MIC_50_ did not differ comparing intervention groups (chlorhexidine and octenidine group) with the control group 12 months after implementation of antiseptic bathing with chlorhexidine or octenidine. Further, we found evidence that ICU day of sample collection was significantly associated with increased proportions of bacterial isolates with MIC ≥ species-specific MIC_50_ for chlorhexidine, but not octenidine_._ Interestingly, bacterial isolates with chlorhexidine MIC ≥ species-specific chlorhexidine MIC_50_ were significantly less likely to originate from blood compared with other sample sites. This observation was not found for octenidine.”

Discussion (line 323ff): “Our GEE model found evidence that length of ICU stay (represented by ICU day of sample collection) was associated with higher proportions of bacteria with increased chlorhexidine MIC. Patients with high length of stay on the ICU most likely received more interventions (e.g. invasive procedures, medication, antibiotic therapy) over longer durations of time. As antibiotic resistance is a known indicator of chlorhexidine susceptibility, longer ICU stays (and in consequence presumably more antibiotic treatment) might have an impact on the chlorhexidine susceptibility of bacterial isolates from clinical samples (37). Further, bacterial isolates with high chlorhexidine MIC were significantly less likely to originate from blood cultures compared with other sources. We can only speculate on the reasons for this finding. One possible explanation might be that bacteria isolated from blood might be less affected by ICU interventions including antiseptic bathings compared with other sample sites, e.g. wounds. However, it is unclear, why we did not see these observations with octenidine.“

Discussion (line 346ff): “Thus, we used species-specific MIC_50_ as threshold to generate binary variables of phenotypic susceptibility for our GEE models. This approach was chosen to apply the same methods for all species and substances (chlorhexidine and octenidine). However, we did not collect all bacterial isolates from clinical samples or a representative sample set from all ICUs participating. Thus, statistical analyses that could be performed to control for potential confounders including cluster-effects were not fully applicable.”

Minor:

7. Please clarify what the proportions of different bacterial species shown in Table 2 represent, i.e., what is the denominator?

Thank you for this comment. We included the column “total” to Table 2 and report the total number of bacterial isolates per species as well as MIC25, MIC50, MIC75 and MIC90 for all bacteria per species. Thus, the total numbers of bacteria per species represent the denominators for the proportions of different bacterial species per study group and study period shown in Table 2. We included the following sentence to the legend of Table2: “Percentages of bacterial isolates per study group and period refer to the total number per species (line 222).”

8. Please correct the following sentence in the conclusion: “However, the observation period of 12 months might be too and the number of bacteria per species and study group too short to draw any final conclusions.”

Done (line 400 ff): However, the observation period of 12 months might be too short and the number of bacteria per species and study group too small to draw any final conclusions.

Reviewer #1: Overall, I find this paper interesting with large pool of isolates and interesting set of data which may be used for other researches. I believe this paper should be published, followed some clarifications.

9. Comment 1: The methods are unclear.

a. It is unclear how did you determine the antiseptic stock solutions concentrations of antiseptic substances (chlorhexidine and octenidine), given the fact that they were 'harvested by wringing out'.

Thank you for this important comment that is identical to the editor’s first comment. The minimal inhibitory concentrations reported in our study were estimations deviated from the concentrations reported on the “ready-to-use antiseptic products” (=extraction solutions) by the manufacturer. Thus, we did not measure but estimated concentrations of the chlorhexidine extraction solution as 2 µg/ml and octenidine extraction solution as 0.08 µg/ml. The contract laboratory used these extraction solutions harvested by wringing out the antiseptic products instead of stock solutions, because the companies did not provide any stock solutions of chlorhexidine and octenidine. To avoid confusion, we used the term “extraction solutions” instead of “stock solutions” for the chlorhexidine / octenidine solutions harvested by wringing out the ready-to-use-products throughout the entire manuscript.

We revised all sections of the manuscript accordingly:

Methods’ section: (line 113 ff).

“Extraction solutions of antiseptic substances (chlorhexidine and octenidine) were harvested by wringing out chlorhexidine-impregnated cloths (Stryker / Sage Products) and octenidine wash mitts (Schülke) provided by the manufacturer. Concentrations of these extraction solutions were estimated to be similar to the concentrations of the ready-to-use products reported by the manufacturers: 2% (20,000 µg/ml) for chlorhexidine and 0.08% (800 µg/ml) for octenidine. Dilutions of antiseptic substances were made in water of standardized water hardness (WSH) according to VAH method 5 [1]. Briefly, bacteria were inoculated in soybean casein digest agar (CSA) at 37°C for 18 – 24h. Concentrations of these bacterial solutions were adjusted to 1.5 – 5.0 x 10^8^ colony forming units (CFU)/ml Subsequently, 150 µl of the respective antiseptic dilutions and 150 µl doubled concentrated soybean casein solutions (CSL) were mixed. Microtiter plates were inoculated with 3 µl bacteria (1:10-dilution in CSL) yielding in a final bacterial concentration of 1.5 – 5.0 x 10^5^ colony forming units (CFU)/ml. Bacteria were inoculated at 37°C for 48 h. The lowest concentration of extraction solutions without bacterial growth (detectable turbidity) was interpreted as minimal inhibitory concentration (MIC) reported in percentage of extraction ~~stock~~ solution. Percentages were converted to estimated (est.) concentrations of extraction solutions in µg / ml. Controls were inoculated with WSH instead of antiseptic dilutions. Determination of bacteriostatic efficacy and suitable neutralizing agents was done according to VAH method 7 [1]. The following reference strains were included to the analysis: *Staphylococcus aureus* ATCC6538 (DSM 799), *Proteus mirabilis* ATCC 14153 (DSM 788), *Klebsiella pneumoniae* ESBL (DSM 16609), *Pseudomonas aeruginosa* ATCC 15442 and *Escherichia coli* NCTC 10538.”

Further, we now explain in the discussion, why we consider our approach being eligible (line 366 ff):

“Further, MIC tests were not performed with pure chlorhexidine and octenidine substances but with extraction solutions harvested by wringing out the antiseptic products. Pure stock solutions were not provided by the manufacturers. In consequence, MIC reported here could only be estimated ~~by~~ from the concentrations reported for the antiseptic products (2µg / ml for chlorhexidine and 0.08 µg/ml of octenidine) and the dilutions (MIC in percentages) derived from of our extraction solutions. This might also limit comparisons to other studies. […] Further, this approach might consider fractions of chlorhexidine and octenidine remaining in the cloths / mitts during the bathing process. This would not be factored in when using pure stock solutions. Thus, we consider our approach being appropriate and even more conservative. However, our estimated MIC might be overestimated and true MICs of bacterial isolates from clinical samples to chlorhexidine and octenidine might be lower.”

b) The production of the Bacterial inoculum is also unclear. It seems that the range of the bacteria CFU is to high 1.5-5.0 108, is it spelling mistake?

The range of bacterial solutions from 1.5 – 5.0 x 10^8 was used according to the VAH method 7 (in German, <https://vah-online.de/files/download/ebooks/eBook_VAH_Methoden_Anforderungen.pdf>[1]. We re-checked this range with the protocol and the contract laboratory conducting all MIC tests for this study. They confirmed this range.

c) Did you meant that the bacterial suspensions were diluted to a final concentration of 10-6 and 10-7? if so, it's need to be better clarify. furthermore, is the bacterial concentration calculated here is before or after the antiseptic and the CSL mixing?

We apologize that we did not explain this method clear enough. We revised the section in the manuscript as follows (line 119ff):

“Briefly, bacteria were inoculated in soybean casein digest agar (CSA) at 37°C for 18 – 24h. Concentrations of these bacterial solutions were adjusted to 1.5 – 5.0 x 10^8^ colony forming units (CFU)/ml ~~in soybean casein digest agar (CSA) at 37°C for 18 – 24h~~. Subsequently, 150 µl of the respective antiseptic dilutions and 150 µl doubled concentrated soybean casein solutions (CSL) were mixed. Microtiter plates were inoculated with 3 µl bacteria (1:10-dilution in CSL) yielding in a final bacterial concentration of 1.5 – 5.0 x 10^5^ colony forming units (CFU)/ml. Bacteria were inoculated at 37°C for 48 h. The lowest concentration of extraction solutions without bacterial growth (detectable turbidity) was interpreted as minimal inhibitory concentration (MIC) reported in percentage of extraction ~~stock~~ solution. Percentages were converted to estimated (est.) concentrations of ~~stock~~ extraction solutions in µg / ml. Controls were inoculated with WSH instead of antiseptic dilutions. Determination of bacteriostatic efficacy and suitable neutralizing agents was done according to VAH ~~2015~~ method 7 (21).”

10. Comment 2: You have a lot of information regarding the isolate origin- e.g blood, urine, tracheal aspirate and liquor- did you looked into differences base on that? If so, show it. It's an interesting data that may contribute largely to current knowledge.

As suggested by the editor and reviewer 3, we included more sophisticated statistical analyses to this study. Within this context, we also included ICU day of sample collection and sample site as variables in the descriptive analyses (please see S4 Table) and as potential confounders in the multivariable analyses (Table 3, line 252). According to our multivariable analysis, bacterial isolates with chlorhexidine MIC ≥ species-specific chlorhexidine MIC50 were significantly less likely to originate from blood compared with other sample sites. This observation was not true for octenidine. We discussed this finding in the manuscript (line 329ff):

“Further, bacterial isolates with high chlorhexidine MIC were significantly less likely to originate from blood cultures compared with other sources. We can only speculate on the reasons for this finding. One possible explanation might be that bacteria isolated from blood might be less affected by ICU interventions including antiseptic bathings compared with other sample sites, e.g. wounds. However, it is unclear, why we did not see these observations with octenidine.“

Reviewer #2: The authors performed a study of phenotypic chlorhexidine and octenidine susceptibility of bacterial isolates from clinical samples obtained before and one year after implementation of a three-armed cluster randomized decolonization trial. The results add to the existing literature addressing the impact of antiseptics on resistance patterns. The study design utilized clinical samples and called for the comparison of bacterial isolates obtained prior to initiation of the study with those obtained at the end of the study. This design results in a population-level comparison and the authors acknowledge the limitations of this type of design compared to that of longitudinal patient sampling. The authors also recognize in detail other limitations of their design in the Discussion. Further, the authors inform the readers that a longitudinal patient-level collection of bacterial isolates was done to study the cutaneous microbiome over time, although it’s unclear whether that study will result in comparisons of resistance markers over time in the three arms. It’s also the case that bacteria found in tracheal aspirates, urine, and CSF may have less of an opportunity to be affected by antiseptics used on the skin. That said, the authors provide the results by bacterial species and clinical sample type, which is a strength of their presentation.

Specific Comments/Questions.

11. Abstract, 3rd paragraph of Introduction. Rather than using the terminology “side effects”, something like “unintended consequences” might be a better choice.

Done. Line 18 in the abstract and line 63 in the introduction.

12. Methods. Why were stock solutions created by wringing out the impregnated cloths and wash mitts rather than purchasing stock solutions or creating stock solutions from a more concentrated liquid source?

This comment is similar to comment 1 by the editor and comment 1 by reviewer 1. Please see the responses to these comments in issue 1 (response to comment 1 by the editor) and issue 9 (response to reviewer 1, comment 1).

Reviewer #3: A 3-arm cluster randomized clinical trial was conducted which aimed to assess the phenotypic susceptibility of bacterial isolates to chlorhexidine or octenidine for antiseptic bathing. The conclusions are unclear.

Major revision:

13. More sophisticated statistical models to test for group by time interactions are called for.

As requested, we included more sophisticated statistical models to test for group by time interactions.

We analyzed results generated in a cRCT. In consequence, we wanted to use multivariable models that consider group by time interactions and potential cluster effects (generalized estimating equation models = GEE models).

For this purpose, we generated a binary variable for the outcomes chlorhexidine / octenidine susceptibility. Unfortunately, epidemiological cut-off values for chlorhexidine were not available for all species (e.g. CoNS), and not available at all for octenidine (18). However, we needed to use the same approach for all bacterial species and substances tested. In consequence, we used species-specific MIC50 as threshold to generate binary variables of phenotypic susceptibility for our GEE models. Species-specific MIC50 were determined in this study (Table 2). We included the new variable to our data set (CHG_>=MIC50, OCT_>=MIC50). Briefly, we categorized all bacterial isolates according to their chlorhexidine and octenidine MIC being ≥ or < the species-specific chlorhexidine and octenidine MIC50 (identified in this study, see Table 2). We generated two categories of bacterial isolates for the variables CHG_>=MIC50 and OCT_>=MIC50: “yes” (= 1; bacterial isolates with chlorhexidine / octenidine susceptibility ≥ species-specific MIC50) or “no” (= 0, chlorhexidine susceptibility of bacterial isolates < species-specific MIC50). Please see S4 Table for the descriptive analyses of these variables and Table 3 for results of the multivariable analyses.

We revised the respective sections in the manuscript as follows:

Methods (line 141ff):

“We generated a binary variable to represent phenotypic chlorhexidine and octenidine susceptibilities of bacterial isolates from clinical samples. All bacterial isolates were categorized according to their chlorhexidine and octenidine MIC being ≥ or < the species-specific chlorhexidine and octenidine MIC_50_ (identified in this study) to generate binary variables. Bacterial isolates were categorized as “yes” (bacterial isolates with chlorhexidine / octenidine susceptibility ≥ species-specific MIC50) or “no” (chlorhexidine susceptibility of bacterial isolates < species-specific MIC50).

In the multivariable analysis, we applied generalised estimating equation (GEE) models to identify an effect of chlorhexidine / octenidine interventions on the susceptibility of bacterial isolates from clinical samples to these substances. We used one GEE model for all species to investigate the susceptibility to chlorhexidine, and another GEE model to investigate the susceptibility to octenidine. The outcomes “phenotypic susceptibilities to chlorhexidine / octenidine” were represented by proportions of bacterial isolates with chlorhexidine or octenidine MIC being ≥ the species-specific chlorhexidine / octenidine MIC_50_. Interaction of study groups (chlorhexidine / octenidine / control) and study periods (baseline / intervention) as well as potential cluster effects were considered in these models [3]. Control group (intervention period) served as reference. Further, ICU day of sample collection and clinical site (blood, tracheal aspirate, urine, wound, or other) were determined as possible confounders and considered in all models. The category “other” as clinical site included e.g. intra-operative samples, intra-abdominal swabs and / or tissue All parameters added one degree of freedom to the model.”

Results (line 252ff):

**“**We used GEE models to investigate the impact of antiseptic bathing with chlorhexidine or octenidine on the chlorhexidine and octenidine susceptibility of bacterial isolates (all species). Characteristics of bacterial isolates (all species) stratified by chlorhexidine or octenidine MIC ≥ species-specific MIC50 (yes / no) are shown in S4 Table. aIRR for the chlorhexidine and the octenidine models are depicted in Table 3. The proportion of bacterial isolates with MIC ≥ species-specific chlorhexidine and octenidine MIC_50_ did not significantly differ between chlorhexidine or octenidine intervention periods compared with the control group (intervention period, Table 3). We detected significant differences between study groups and study periods with our GEE models (Table 3). However, these differences seemed to be independent from antiseptic bathing. In fact, aIRRs showed that the proportions of bacterial isolates with chlorhexidine / octenidine MIC ≥ species-specific chlorhexidine / octenidine MIC_50_ did not differ comparing intervention groups (chlorhexidine and octenidine group) with the control group 12 months after implementation of antiseptic bathing with chlorhexidine or octenidine. Further, we found evidence that ICU day of collection was significantly associated with increased proportions of bacterial isolates with MIC ≥ species-specific MIC_50_ for chlorhexidine, but not octenidine_._ Interestingly, bacterial isolates with chlorhexidine MIC ≥ species-specific chlorhexidine MIC_50_ were significantly less likely to originate from blood compared with other sample sites. This observation was not true for octenidine”.

Discussion (line 323ff):

Our GEE model found evidence that length of ICU stay (represented by ICU day of sample collection) was associated with higher proportions of bacteria with increased chlorhexidine MIC. Patients with high length of stay on the ICU most likely received more interventions (e.g. invasive procedures, medication, antibiotic therapy) over longer durations of time. As antibiotic resistance is a known indicator of chlorhexidine susceptibility, longer ICU stays (and in consequence presumably more antibiotic treatment) might have an impact on the chlorhexidine susceptibility of bacterial isolates from clinical samples (Quelle Koljalg). Further, bacterial isolates with high chlorhexidine MIC were significantly less likely to originate from blood cultures compared with other sources. We can only speculate on the reasons for this finding. One possible explanation might be that bacteria isolated from blood might be less affected by ICU interventions including antiseptic bathings compared with other sample sites, e.g. wounds. However, it is unclear, why we did not see these observations with octenidine.

We also discussed limitations of our multivariable analyses (line 346ff):

“Further, epidemiological cut-off values for chlorhexidine were not available for all species (e.g. CoNS), and not available at all for octenidine [4]. Thus, we used species-specific MIC_50_ as threshold to generate binary variables of phenotypic susceptibilities to chlorhexidine and octenidine for our GEE models. This approach was chosen to apply the same methods for all species and substances (chlorhexidine and octenidine). However, we did not collect all bacterial isolates from clinical samples or a representative sample set from all ICUs participating. Thus, statistical analyses that could be performed to control for potential confounders including cluster-effects were not fully applicable.”

Further, we rephrased the conclusion and hope that it becomes more clear now (line 398ff):

“We found no evidence for reduced chlorhexidine or octenidine susceptibilities of bacterial isolates from clinical samples in ICUs after implementation of daily patient bathing with these antiseptics. However, the observation period of 12 months might be too short and the number of bacteria per species and study group too small to draw any final conclusions. In consequence, results must be interpreted with caution.“

Minor revisions:

14. 1- Table 1, title: In statistical terms, stratification refers to a process used during randomization. Consider replacing this term in the title.

Thank you for this comment. We revised the title of table 1 accordingly (line 204f):

“Table 1: Characteristics of ICU-attributable bacterial isolates from clinical samples included in the analyses (n = 790) in total and reported by study group and period.“

15. 2- State and justify the study’s target sample size with a pre-study statistical power calculation. The power calculation should include: sample size, alpha level (indicating one or two-sided), minimal detectable difference and statistical testing method.

Thank you for this comment. As requested, we performed a power calculation including sample size, alpha level (two-sided), minimal detectable difference and statistical testing method (line 161ff):

“Initial sample size calculation of this cRCT was done for the primary outcome CLABSI [5]. However, we performed a power calculation for the outcomes “phenotypic susceptibilities to chlorhexidine / octenidine” represented by proportions of bacterial isolates with chlorhexidine or octenidine MIC being ≥ the species-specific chlorhexidine / octenidine MIC_50_. The latter were identified in this study (Table 2). We assumed that at the end of the intervention period, 35% of isolates were ≥ MIC_50_ in the control group (routine care) compared to 65 % in the intervention group (antiseptic bathing with chlorhexidine or octenidine). Further, we assumed a ratio of 1:1 between the groups. With individual randomization, a sample size of 70 bacterial isolates in each group would have been required to show the difference with a power of 80%, and a two-sided type 1 error of 0.025. Adjusting for cluster effects with 13 clusters per arm and an intracluster correlation coefficient of 0.1 (adapted from literature [2]), we must have included 101 bacterial isolates per arm. Multiple comparisons to the routine care group were considered by a two-sided type 1 error of 0.025. “

16. 3- To assist in the review process, add line numbering to the document.

Thank you for this comment. We revised the manuscript accordingly and included lines to the manuscript with track changes and the clean version. All lines reported in this rebuttal letter refer to the manuscript version with track changes.

References

[1] Verbund für Angewandte Hygiene e.V. (VAH), Anforderungen und Methoden zur VAH-Zertifizierung chemischer Desinfektionsverfahren, Desinfektionsmittel-Kommission im VAH, 2019.

[2] A. Donner, N. Birkett, C. Buck, Randomization by cluster. Sample size requirements and analysis, Am J Epidemiol 114(6) (1981) 906-14.

[3] S.L. Zeger, K.Y. Liang, P.S. Albert, Models for longitudinal data: a generalized estimating equation approach, Biometrics 44(4) (1988) 1049-60.

[4] G. Kampf, Acquired resistance to chlorhexidine - is it time to establish an 'antiseptic stewardship' initiative?, The Journal of hospital infection 94(3) (2016) 213-227.

[5] L.A. Denkel, F. Schwab, J. Clausmeyer, M. Behnke, J. Golembus, S. Wolke, P. Gastmeier, C. Geffers, Effect of antiseptic bathing with chlorhexidine or octenidine on central-line associated bloodstream infections in intensive care patients: a cluster-randomised controlled trial, Clinical microbiology and infection : the official publication of the European Society of Clinical Microbiology and Infectious Diseases (2022).
